# Supplementary material for: Blastocystis colonization and associations with population parameters in Thai adults
Source: PLoS Negl Trop Dis. 2024 Jul 9;18(7):e0012292. doi: 10.1371/journal.pntd.0012292 (PMC11259296; doi:10.1371/journal.pntd.0012292)
Supplement: Attachment — Submitted filename: PLOS_NTD_rebuttal.docx [file pntd.0012292.s003.docx]

**All reviewers and editor:** Given the comments of the reviewers and considering the size of the dataset and the population parameters, we brought in our team an epidemiologist who rerun the analyses and performed additional tests. Details of the methodology and results obtained are highlighted in the text

**Reviewer #1**
In this manuscript Jinatham et al. describes the occurrence and genetic diversity of the protist of uncertain pathogenicity Blastocystis in an apparently healthy adult population from rural and suburban settings in Thailand. The authors also appraised whether BMI and other sociodemographic variables (age, gender, location) were associated with an increased likelihood of Blastocystis carriage. Detection of the protist was accomplished by molecular (nested-PCR and qPCR) methods, whereas genotyping was conducted by Sanger sequencing of the amplification products obtained by both PCR methods. A thorough statistical analysis involving logistic regression and multiple correspondence analyses were also carried out to assess associations among risk factors and Blastocystis carriage. Overall, the manuscript provides some useful prevalence and molecular data from an endemic area where relatively little information is available on the epidemiology of Blastocystis.

*** We thank the reviewer for their positive comments ***

However, the study has some design and methodological limitations that must be taken into account by the Authors when interpreting results and raising conclusions.

*** We are thankful to the reviewer for this comment. We brought in the team an epidemiologist (Eleni Michalopoulou) who conducted the analysis and assisted with phrasing conclusions (please see below) ***

I would also like to bring to the Authors´ attention the convenience of numbering pages and lines of the manuscript to facilitate the review process.

*** Indeed. The authors apologize for this oversight. The revised version has numbered pages and lines ***

1.     Sample collection: please indicate the inclusion/exclusion criteria followed when recruiting participants for the study. In my opinion this is a critical step any study design attempting to correlate the presence of a microorganism with the outcome of the infection or any other variable considered.

*** The criteria are now included in the materials and methods section, study sites and sample collection subsection ***

2.      Following this very same line of reasoning, the authors focused on the association Blastocystis vs. BMI. Did they check for the presence of other enteric parasites that might be acting of confounders of the obtained results? If this information is available, I would encourage the Authors to mention it in the manuscript and to reinterpret their results under the light of potential co-infections (expected at high rated in rural areas from endemic countries such as Thailand. If this information is not available, this should be stated as a limitation of the survey.

*** The plan was indeed to screen for other parasites, most notably Giardia and Cryptosporidium, however this has not happened yet. This is now mentioned in the methodology section and also it is stated as a limitation ***

Another important issue that must be acknowledged by the Authors is that BMI data was unavailable for 36% of the participants. This, together with the fact that sample size is not particularly high, might have compromised the quality and robustness of the obtained results.

*** Yes, missing data is always a concern for the precise reasons that the reviewer pointed out. To that end, before performing the statics, the power of the test was calculated (power=0.8135; α=0.05) which indicated that sample size was sufficient and the conclusions drawn have merit for this specific population. This has now been added to the results section ***

3.      In this regard, it is also unclear to me why the Authors restricted the study to individuals of adult age. If Blastocystis is mainly a commensal (rather than a pathogen) microorganisms in most instances, we should expect increased prevalence rates through lifetime as once the protist colonizes the gut it remains there as part of the microbiome for prolonged period of time. Information from longitudinal studies conducted in human populations in Ireland (see PMID 25077936) and Spain (see PMID 36282323) and in animal populations in USA (see PMID 37264466) seem to support this notion. By focusing only on adult individuals (more likely to carry Blastocystis) I wonder whether the potential effect of primary colonization at younger ages (e.g., paediatric populations) and its effect in BMI is missed. I invite the Authors to explore this possibility more in depth and comment on it.

*** We wholeheartedly agree with the reviewer on the importance of pediatric populations, especially background pediatric populations examined at various timepoints. We feel that this type of study (involving children) should be conducted separately as was the case with the studies in Spain (PMID 36282323) and Ireland (PMID 29397054) for example. In that vein, we have conducted a children-based study in Thailand at two time points and published this work separately (PMID: 37185159) ***

Regarding potential correlation between Blastocystis and obesity, please also see PMIDs 36358463, 35956387, and 34650200. These references can contribute to enrich the quality and interest of the results obtained in this survey.

***We thank the reviewer for pointing out these very interesting papers! We have added these and modified the discussion pertaining to BMI***

4.      In the Material and Method sections, it is very unclear to me what was the diagnostic algorithm used in the present study. Authors used simultaneously nested-PCR and qPCR. Considering that the latter has more sensitivity and, judging the results obtained here, much better specificity, it seems more reasonable using qPCR as screening method and nested-PCR for genotyping purposes. What was the rationale behind this decision and which benefits were obtained from it?

*** The purpose of using both PCR and qPCR was indeed as the reviewer mentioned. We have now added the rationale behind using these approaches in the materials and methods section under the molecular detection subheading ***

5.      Also, the molecular part of the Material and Methods section is poorly structured and described. I would recommend following this scheme using independent sub-sections:
•       DNA extraction and purification
•       qPCR protocol
•       nested-PCR protocol, including gel electrophoresis and imaging
•       Sequencing and sequence analyses
Please note that information provided in these sub-sections should suffice to allow the interested reader to reproduce the experiments without checking primary sources.

***The methods section was rearranged. We did not add detailed instructions of manufacturer’s protocols as these can be easily accessed online and their inclusion is not standard practice ***

6.      No GenBank accession numbers are provided, so I was unable to assess the quality of this material. This task must be conducted in a potential second round of review, particularly for less common genetic variants such as ST23. Please provide this information.

*** The data for the ST23 subtypes is already available (OL351649–OL351797). This is mentioned in the methodology section. The rest of the data was submitted and will be released upon publication. The accession numbers are PP728964-PP729011, PP729403-PP729456 and PP731515-PP731535. A list of the submitted sequences is provided for the reviewers’ perusal ***

7.      Discussion section, first paragraph: when comparing results from similar studies conducted in Thailand, I think it is important to provide information regarding the populations surveyed and the detection methods used to enable direct data comparison. Please amend.

*** We agree with the reviewer. Methodologies used and differences in populations and demographic factors considered definitely play a role. These points were included in the discussion ***

8.      Discussion section: Authors correctly point out to the animal contamination to explain the origin of ST10 and ST23, but in my opinion they should do the same for ST7 as pigs are recognized hosts for this Blastocystis genetic variant. Please comment on this important matter to highlight the importance of waterborne/foodborne transmission and to give also a One Health perspective to the discussion of this information. Regarding the virulence/pathogenicity of ST7, I think (I am talking from the top of my head and might be wrong) that most the evidence for this statement come form in vivo assays with experimentally infected model animals conducted by Kevin Tan´s group. Please check. If true, this information should be clearly indicated in the manuscript.

*** Blastocystis continues to enthrall its “audience”. It really is a mystery. ST7 is carried by birds worldwide, yet when it comes to humans, it is mainly found in Asia. We see a similar thing with ST4. It is carried by rodents worldwide, yet mainly found in humans living in Europe. A similar situation seems to be emerging with ST10. A paragraph has been added discussing transmission of ST7 and linking it with our previous such work in the area.

9.       The Authors must include at the end of the Discussion section a whole paragraph stating the limitations of the study (sample size, potential bias associated to the selected population under study, limited BMI data, absence of co-infection data, etc.). Please be as thorough and precise as possible.

***We included a limitations paragraph in the end of discussion. Absence of coinfection data was noted in the methodology. ***

Minor issues
1.      Decima figures: Authors use two decimal figures through the text. I believe that a single one is precise enough (e.g., 67.23% should be better expressed as 67.2%. Please modify through text and Tables).

***Agreed. This was changed throughout except in the case of statistical tests and confidence intervals ***

2.      Introduction section, second paragraph: SSU (as all gene abbreviations) should be italicised. Amend through the whole manuscript.

*** SSU was italicized throughout the manuscript ***

3.      Introduction section, second paragraph. Please note that at present 42 valid Blastocystis STs are considered valid. ST45 and ST46 have been recently reported in Australian marsupials (see Koehler et al., 2024 [https://doi.org/10.1016/j.ijppaw.2023.100902](https://doi.org/10.1016/j.ijppaw.2023.100902)). Also, please note that current references 21-24 do not cover all Blastocystis STs reported. These should also include:
•       ST32: PMID 34504891
•       ST35-38: PMID 36677338
•       ST39: PMID 36932438
•       ST40: PMID 37355198
•       ST41: PMID 37195413
•       ST42-44: PMID 37658622
•       ST45-46: [https://doi.org/10.1016/j.ijppaw.2023.100902](https://doi.org/10.1016/j.ijppaw.2023.100902)
Please amend and update this info with the primary references.

*** We do not disagree with the reviewer. At some point there will be so many subtypes that the primary references will occupy several lines. We have now included all primary references***

4.      Introduction section, second paragraph: please note that 16 (not 14) Blastocystis STs have been reported in humans. These include ST1-ST10, ST12, ST14, ST16, ST23, ST35, and ST41. Please see PMID 27034056, 32932661, 34956115, 33467077, 34356524, 37195413, and 37658622). Please amend and update this info with the primary references.

***Amended***

5.      Cloning subsection: pLUG-Prime® (in superscript). In my opinion, the last three sentences of this section are better suited for the Results section.

*** The ® has been removed, as trademark symbols are not publish in this journal. The sentences mentioning the number of products cloned were moved to the results ***

**Reviewer #2**

I read with great interest the manuscript by Jinatham et al who conducted a modestly sized but well-designed epidemiological study on the occurrence, subtypes and predictors of Blastocystis sp. in Thailand, focusing on peri-urban / rural differences, and associations with BMI.
The design is sound and relevant to the research question. The methods (with one exception) are appropriate and supported by the literature and the experience of the author team. The results are clearly presented, with the exception of one figure.
The interpretation of the results in Discussion is interesting, mostly reads well and is sober (except for some problems with the interpretation of statistical significance or lack thereof).

*** Reviewer one also commented on the statistics. We brought an epidemiologist in, who analyzed the data and assisted with interpretations (please see highlighted tect)***

I would like to suggest two points that seem important to me. Addressing them would improve the quality of the manuscript.
(a) Detection methods should be discussed so that the reader understands their purpose. Readers may be confused as to why two PCR methods were used. The rationale behind the combination needs to be briefly explained to readers outside the Blastocystis community.

*** Reviewers one and three also made the same point. The purpose of using both PCR and qPCR was indeed as the reviewer mentioned. We have now added the rationale behind using these approaches in the materials and methods section under the molecular detection subheading ***

(b) I found the method of subcloning the (q)PCR products rather cumbersome and definitely underpowered to detect a mixed infection. While I fully understand that the use of NGS for subtyping a benign parasite in a research setting in a middle-income country may be problematic. I find it difficult to understand why only two colonies were picked after cloning the product. Given that a mixed infection can be detected on Sanger chromatograms from about 20% admixture of the weaker signal, we can simplify to say that the odds of detecting the weaker subtype is 1:4. Two colonies is definitely not enough; even if the signal ratio were 1:1, it would still be too few... The costly thing is the transformation and selection - taking only two colonies is a bit of waste of resources.

*** The reviewer has phrased the difficulties encountered in an LMIC comprehensively and we thank the reviewer for their understanding. We agree that screening two colonies is absolutely not enough to detect mixed infection. In fact, the intention was not to detect mixed infections, but to obtain a clean sequence. So we screened two colonies to maximize the chances of getting said clean sequence. We have added this clarification in the methodology section under the cloning subheading ***

The statement "Altogether 88.75% (142/160) of Blastocystis-positive individuals carried a single subtype" is too optimistic, as the method does not have sufficient power to detect mixed subtypes. 

*** We agree with the reviewer, the sentence was rephrased to: “Altogether, we detected a single subtype in 88.8% (142/160) of *Blastocystis*-positive individuals, whereas a mixed-subtype (mixST) infection was detected in 3.8% (6/160)”. We have also added this point in the discussion section***

Minor points:
The authors used quantitative PCR, but we do not see any information on the quantity - the SYBR green gives approximate quantities at least in the early cycles.

*** Even though this approach can be used for quantitative purposes as the reviewer pointed out, we used it solely for the purpose of screening the samples. All reactions were run in gels and if the desired band was present, the product was purified and sequenced. This is now mentioned in the methodology section ***

Unclear wording: reconsider whether the information in the sentence "However, 56 PCR products were false positives..." should be presented in the paragraph on Blastocystis prevalence.

*** Amended as per reviewer’s suggestion ***

"more at risk of carrying Blastocystis"; " age seems to be a risk factor for the
positivity of Blastocystis". Well, this is testing for associations. It gives odds ratios, I would not call it risk, because the protist may be beneficial rather than harmful as the authors rightly point out in the Introduction. Consider rewording to reflect this. 

*** statistics parts in all sections of the manuscript have been reworded (please see highlighted text) ***

Table 2: The presentation of "No. (%) negative" is rather surprising. We can subtract the positivity from 100% without having to present another column.

*** We would like to keep both positive and negative columns, though now tables are displayed differently ***

I am not very happy with Figure 2. It is not self-explanatory. The totals differ because of 1) an uneven number of individuals tested in the category, 2) uneven positivity rates, 3) an uneven/noisy distribution of subtypes. The number of subtypes in the subcategories is very difficult to read from the stacked plots. I am afraid that this figure could serve as a textbook example of a confusing plot. Please consider conveying the information in a clearer way (not stacking? table? numerical labels? completely different format?).

***After careful consideration, we replaced figure two with a table, which is indeed more informative ***

Figure 3 is better, but still no totals are given (is it subject-wise, positivity-wise or other percentage?). Pie charts are sometimes frowned upon in the scientific community. In any case, avoid false precision (3.78%, 1.89%... 38.08%).

*** The figure was modified. Percentages have only one significant digit, and totals are shown ***

I am not convinced that the MCA adds anything to the multiple logistic regression model. It seems to me that it only adds unnecessary complexity without showing new facts.

*** We agree with the reviewer. The MCA was removed ***

I suggest that the authors refrain from claiming priority ("this is the first report on both the prevalence..."). Admittedly, Blastocystis is not a deadly pathogen and is understudied, so most studies are first in one way or another. We do not need "first" epidemiological studies, we need a worldwide exploration and replication of things found elsewhere to learn more äbout the epidemiology of this protist.

*** This is very much so. The “first…” claim was removed ***

"The higher prevalence of Blastocystis in obese rather than lean subjects was surprising." Where in the analysis do we see such a difference? I don't see one! It is NOT a significant difference, so it is NOT a difference! Please refrain from speculations on what would happen if there were more subjects.

***We agree. The new addition to the team performed analyses and explored models and the results have changed (see the highlighted text)

The journal's data sharing policy seems to require that all data be shared. Please share the table with metadata (the one for the logistic regression).

***We exported the metadata sheet from Stata and provide it as a supplementary file***

Reviewer #3: (No Response)

# Reviewer Comments to Author

First of all, I want to thank to all of the authors of the manuscript. *Blastocystis* is a very common enteric protist parasite in humans and animals, and its importance has been increasing in recent years. Although there is still debate about whether Blastocystis is a pathogen or not, mechanisms that have been demonstrated for some subtypes, especially ST7, are mentioned. Also, an important study examining the distribution and potential relationships between the carriage of Blastocystis in rural/peri-urban Thai adults.

*** We thank the reviewer for their positive comments***

It would be helpful if the authors explain some points.

Some of those;

Material and Methods;

- Although nested PCR is used to investigate Blastocystis DNA, what is the purpose of studying all samples again with qPCR?

*** We used qPCR for detection and PCR for subtyping. We have now added the rationale behind using these approaches in the materials and methods section under the molecular detection subheading***

- In addition, it would be much more appropriate to provide information about which primers were used in the DNA sequencing method of all positive samples and whether single- or double-direction DNA sequencing was performed.

*** This information is now included in the methodology (see highlighted text under qpcr and pcr product purification and sequencing)***

- Constructing a phylogenetic tree of Blastocystis subtypes in rural and urban areas may make the study more powerful.

***We refrained from constructing a phylogenetic tree in this study as we wanted to emphasize other aspects***

Results;

Are the false positive results of the 56 PCR products in Sanger sequencing the samples detected positive in nested PCR?

***Yes after running the gel we visualized bands of the correct size, which we purified and ended up with non-Blastocystis amplicons. We think that perhaps this is indeed because of two reactions in the nested pcr***

How do you interpret the fact that people living in rural areas have a significantly higher risk of carrying *Blastocystis*? There are situations such as environmental contamination, socio-economic situation, or a higher risk of zoonotic transmission.

***We very much agree with the reviewer. In the case of this study and our previous studies in the area cyclic of subtypes in the environment and various hosts seems to be a very important factor. We now discuss this in the text***

It may be important to evaluate whether there is a difference between Blastocystis subtypes, especially ST3 alleles, in people living in rural and urban areas.

***We agree with the reviewer but we feel that this endeavor should be left for a later study***
