# Supplementary material for: Blastocystis colonization and associations with population parameters in Thai adults
Source: PLoS Negl Trop Dis. 2024 Jul 9;18(7):e0012292. doi: 10.1371/journal.pntd.0012292 (PMC11259296; doi:10.1371/journal.pntd.0012292)
Supplement: Attachment — Submitted filename: Reviewer Comments to Author_28.01.2024.docx [file pntd.0012292.s002.docx]

# Reviewer Comments to Author

First of all, I want to thank to all of the authors of the manuscript. *Blastocystis* is a very common enteric protist parasite in humans and animals, and its importance has been increasing in recent years. Although there is still debate about whether Blastocystis is a pathogen or not, mechanisms that have been demonstrated for some subtypes, especially ST7, are mentioned. Also, an important study examining the distribution and potential relationships between the carriage of Blastocystis in rural/peri-urban Thai adults.

It would be helpful if the authors explain some points.

Some of those;

Material and Methods;

- Although nested PCR is used to investigate Blastocystis DNA, what is the purpose of studying all samples again with qPCR?
- In addition, it would be much more appropriate to provide information about which primers were used in the DNA sequencing method of all positive samples and whether single- or double-direction DNA sequencing was performed.
- Constructing a phylogenetic tree of Blastocystis subtypes in rural and urban areas may make the study more powerful.

Results;

Are the false positive results of the 56 PCR products in Sanger sequencing the samples detected positive in nested PCR?

How do you interpret the fact that people living in rural areas have a significantly higher risk of carrying *Blastocystis*? There are situations such as environmental contamination, socio-economic situation, or a higher risk of zoonotic transmission.

It may be important to evaluate whether there is a difference between Blastocystis subtypes, especially ST3 alleles, in people living in rural and urban areas.
